# Targeted sampling of cementum for recovery of nuclear DNA from human teeth and the impact of common decontamination measures

**DOI:** 10.1186/2041-2223-4-18

**Published:** 2013-10-18

**Authors:** Denice Higgins, John Kaidonis, Grant Townsend, Toby Hughes, Jeremy J Austin

**Affiliations:** 1 Australian Centre for Ancient DNA, School of Earth and Environmental Sciences and Environment Institute, University of Adelaide, South Australia 5005, Australia; 2 School of Dentistry, University of Adelaide, South Australia 5005, Australia

**Keywords:** Cementum, Sodium hypochlorite decontamination, Forensic DNA typing, Skeletal remains, Teeth

## Abstract

**Background:**

Teeth are a valuable source of DNA for identification of fragmented and degraded human remains. While the value of dental pulp as a source of DNA is well established, the quantity and presentation of DNA in the hard dental tissues has not been extensively studied. Without this knowledge common decontamination, sampling and DNA extraction techniques may be suboptimal. Targeted sampling of specific dental tissues could maximise DNA profiling success, while minimising the need for laborious sampling protocols and DNA extraction techniques, thus improving workflows and efficiencies. We aimed to determine the location of cellular DNA in non-degraded human teeth to quantify the yield of nuclear DNA from cementum, the most accessible and easily sampled dental tissue, and to investigate the effect of a common decontamination method, treatment with sodium hypochlorite (bleach).

We examined teeth histologically and subsequently quantified the yield of nuclear DNA from the cementum of 66 human third molar teeth. We also explored the effects of bleach (at varying concentrations and exposure times) on nuclear DNA within teeth, using histological and quantitative PCR methods.

**Results:**

Histology confirmed the presence of nucleated cells within pulp and cementum, but not in dentine. Nuclear DNA yields from cementum varied substantially between individuals but all samples gave sufficient DNA (from as little as 20 mg of tissue) to produce full short tandem repeat (STR) profiles. Variation in yield between individuals was not influenced by chronological age or sex of the donor. Bleach treatment with solutions as dilute as 2.5% for as little as 1 min damaged the visible nuclear material and reduced DNA yields from cementum by an order of magnitude.

**Conclusions:**

Cementum is a valuable, and easily accessible, source of nuclear DNA from teeth, and may be a preferred source where large numbers of individuals need to be sampled quickly (for example, mass disaster victim identification) without the need for specialist equipment or from diseased and degraded teeth, where pulp is absent. Indiscriminant sampling and decontamination protocols applied to the outer surface of teeth can destroy this DNA, reducing the likelihood of successful STR typing results.

## Background

**Figure 1:**
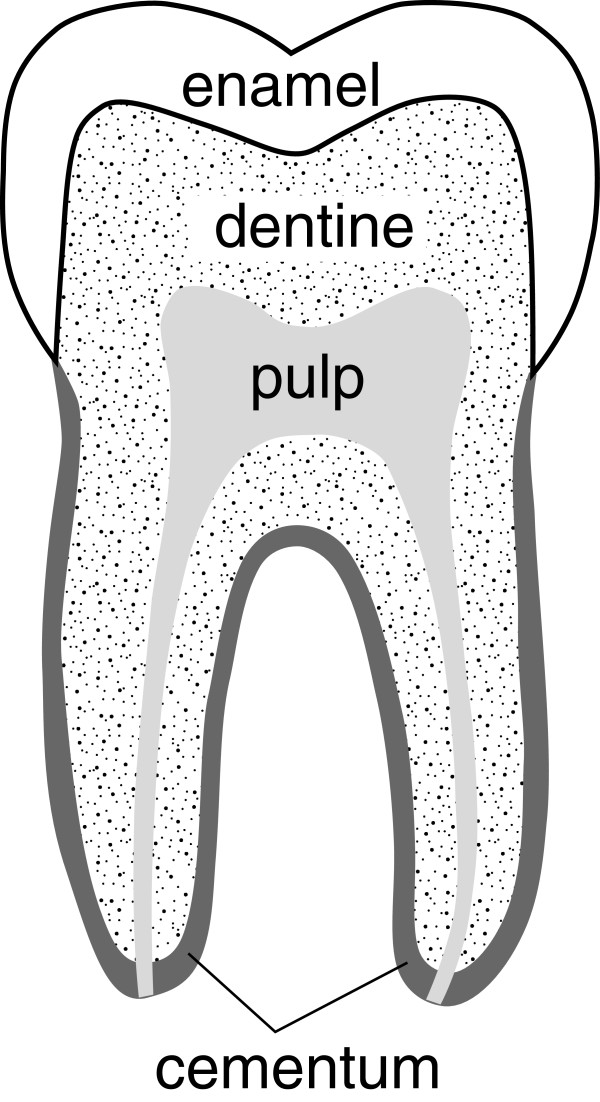
Diagrammatic representation of a human molar identifying the different regions and tissues.

In forensic cases involving unidentified bodies often the only sources of DNA for identification are the calcified tissues - bones and teeth. Teeth are a valuable source of DNA [1,2] due to their unique composition and location in the jawbone both of which provide protection from microorganisms and environmental factors responsible for postmortem decay. Surprisingly, little is known about the location of, nor antemortem and postmortem changes in, DNA in teeth. While pulp is recognised as the richest source of DNA in healthy fresh teeth [3] its value is decreased in life by age [4] and dental disease and in death by postmortem degradation (Figure 1). In an ideal dry postmortem environment pulp may mummify [5] and persist for extended periods but in a moist environment putrefaction rapidly leads to complete destruction [6]. The hard tissues of the tooth - cementum, dentine and enamel (Figure 1) - are more resistant to postmortem decay but targeted sampling of these tissues for nuclear DNA has not been examined in any depth.

MtDNA and nuclear DNA have been obtained from dentine [3,7-10] but the success rate for short tandem repeat (STR) typing of nuclear DNA is variable [8] and the quantity of nuclear DNA available from dentine is negatively affected by age of the individual and dental disease [9], suggesting a strong relationship between the presence/absence of pulp and recovery of DNA from dentine. In contrast, cementum has been shown to be in some instances a better source of mtDNA than dentine (at least in degraded and ancient teeth) [3,7] and yields of nuclear DNA from cementum are not negatively affected by dental disease or chronological age [9,11].

Recovery of DNA from teeth is complicated by mineralisation of the tissues requiring specialised sampling equipment, additional dedicated laboratory space and modified DNA extraction protocols. The major mineral and organic components of teeth - hydroxyapatite (predominantly calcium) and collagen - vary across different dental tissues, with enamel being 96% mineral, dentine 70% mineral and 20% collagen, and cementum 45% mineral and 30% collagen. Pulp is largely cellular and has no mineral content. Both calcium and collagen have been shown to be inhibitors of polymerase chain reaction (PCR) amplification [12] and as such their co-extraction needs to be minimised. A further complication for sampling of mineralised tissues is the fact that an intimate relationship between DNA and hydroxyapatite has been identified in post-mortem samples [13,14] necessitating demineralisation of these tissues for maximum recovery of DNA [15]. Complete demineralisation of bones and teeth using ethylenediaminetetraacetic acid (EDTA) has been shown to improve DNA recovery [15], but requires larger extraction volumes increasing costs and reducing possibilities for automation. EDTA is also a PCR inhibitor so needs to be removed along with calcium and collagen prior to downstream processes.

Improved knowledge of the location of DNA in teeth would facilitate targeted sampling of tissues known to contain nuclear DNA over a range of postmortem intervals and environmental conditions. This could reduce the need for complex and laborious sampling and grinding protocols (including cutting and grinding equipment and dust extraction), allow for smaller sample volume, less calcium and collagen, and reduced dependence on EDTA. Targeted sampling of pulp tissue, which would negate these issues, has been reported via drilling through the crown or by tooth sectioning [16]. However, this does not always have a positive outcome as determining the presence/condition of any pulp tissue prior to sampling is not possible. Cellular cementum may be an important source of nuclear DNA particularly in diseased and degraded teeth where pulp tissue is reduced or absent and therefore the likelihood of retrieving DNA from pulp and dentine is reduced [9]. However, cementum is rarely targeted for DNA analysis and potentially may be removed or damaged during decontamination and sampling.

Prior to sampling, teeth are frequently subjected to decontamination processes aimed at removing exogenous DNA, environmental contaminants and micro-organisms [17]. Decontamination methods vary and include the following, either individually or in combination: removal of the outside layer of the tooth by grinding or sanding [17]; washing/soaking in bleach [18]; washing/soaking in ethanol or in hydrogen peroxide [19]; and exposure to ultra violet (UV) light [20]. Decontamination techniques, mostly designed to destroy exogenous DNA, have an unknown effect on endogenous DNA, and this may be particularly acute for cementum, which forms the outer surface of the root.

The most commonly reported decontamination methods are removal of the outer surface and washing/soaking in bleach of various concentrations for varying time periods [17]. Studies examining the impact of bleach on bone suggest that endogenous DNA is relatively well protected, possibly due to adsorption to hydroxyapatite [17] or entrapment within mineral aggregates [14,21]. However, it is unknown whether DNA binds to tooth mineral and, if so, at what point during postmortem decay this occurs. Studies examining the relationship between DNA and mineral have been performed on bone [14,21], which is structurally and biochemically distinct from tooth negating the reliability of extrapolation of this data to teeth. No studies have explicitly examined teeth.

The aim of this study is to examine how the efficiency of tooth sampling protocols and the success of DNA profiling might be improved through specific targeting of tissues containing nucleated cells. We confirm the location of nucleated cells in fresh teeth, quantify the yield of nuclear DNA from tooth cementum, and examine the effects of bleach on the nucleated cells/nuclear DNA content of cementum. We show that cementum is a valuable and easily accessible source of DNA in teeth that by virtue of its location is at risk of damage from common decontamination methods.

## Methods

One molar tooth was collected from each of 106 volunteer donors along with a blood sample for reference DNA. All work was undertaken under the ethical guidelines and approval from the Research Ethics and Compliance Committee of The University of Adelaide (H-134-2009). Teeth were removed under sterile conditions by registered specialist dental surgeons and placed directly into individually labelled sterile containers.

### Initial histological examination

A randomly selected subset of teeth (*n* = 4) was formalin fixed (neutral buffered 10% formaldehyde) for 72 h and demineralised in 10% EDTA at a pH of 7.4. Total demineralisation was confirmed by radiographic analysis. Teeth were embedded in paraffin wax and sliced in 7 μm sections, slide mounted and stained with haematoxylin and eosin. Haematoxylin binds to chromatin in the DNA/histone complex, staining nuclear material a dark violet colour. The location of nuclei in teeth sections was determined by examination under 100×, 200× and 400× magnification using a compound light microscope (Leica Microsystems, Germany).

### DNA yield from cementum

A further randomly selected subset of teeth (*n* = 66) was cleaned by gentle curettage with a dental scaler to remove soft tissue remnants and blood, and then wiped with DNA-free saline. Cementum samples, in the form of a coarse powder, were scraped from each tooth using a new disposable scalpel blade for each sample. Care was taken to avoid sampling deep concavities or very tight spaces between roots as these sites can retain soft tissue remnants. All samples collected weighed between 15 and 50 mg, dictated by the availability of tissue and the conservative nature of sampling. All equipment and workbenches were cleaned with 4% sodium hypochlorite before and after sampling each tooth.

All pre-PCR work was undertaken in a dedicated pre-PCR laboratory housed in a separate building to the post-PCR laboratory. DNA extractions were performed using QIAmp DNA Investigator kits (Qiagen, Ilden, Germany), following the manufacturer’s instructions for bones and teeth, including the use of carrier RNA. As per this protocol the cementum powder was initially lysed overnight with buffer ATL and Proteinase K at 56°C without prior decalcification. Reference samples were extracted in the same fashion but on a separate day. The final elution volume for each sample was 60 μL. Extraction blanks were included with every set of extractions, one for every three teeth. Extracts were stored at 4°C until quantification and STR profiling.

DNA extracts were quantified using Quantifiler™ Human DNA Quantification Kit, (Applied Biosystems, Foster City, CA, USA) on an ABI PRISM^®^ 7000 Sequence Detection System for real-time PCR (Applied Biosystems, Foster City, CA, USA). Negative and positive controls and seven standards were included in duplicate on each run as directed by the manufacturer. All extraction blanks were quantified. Nuclear DNA concentration was determined using the comparative CT method with unknown samples compared to a standard curve with a range of 0.05 ng/μL to 200 ng/μL. DNA yields were converted to nanograms of DNA per milligram of cementum to allow direct comparison between all samples.

STR profiling of samples and references was performed using Amplf*l*str ProfilerPlus™ (Applied Biosystems, USA). Reactions were performed in 25 μL reaction volumes, consisting of 9.6 μL reaction mix, 5 μL primer mix, 0.4 μL AmpliTaq Gold™ and 10 μL of DNA extract. Cycling was performed on a 9700 GeneAmp Cycler. Amplification parameters consisted of an initial denaturation step at 95°C for 10 min., followed by 28 cycles of 94°C for 60 s, 59°C for 60 s, 72°C for 60 s and a final extension step at 60°C for 45 min.

Capillary electrophoresis was performed on a 3130xl Genetic Analyzer and genotypes analysed using Genemapper ID v3.2.1. A minimum threshold of 50 relative fluorescence units (RFU) was used for calling alleles and the profiles generated were compared to their respective reference.

Compilation of quantification data and descriptive statistics were undertaken in Excel (Microsoft, USA). Inferential statistical tests were performed using SAS STAT software. Statistical significance was set at *P* <0.05 for all tests unless otherwise indicated. The distribution of DNA yield from cementum was examined for normality and significant outliers, and was found to be substantially positively skewed. The data were subsequently log-transformed for analysis. A random effects mixed linear model of DNA yield was fitted to the log data using the 'Mixed’ procedure in SAS STAT software. The model included the fixed effect of sex and the covariate age, as well as the interaction between sex and age. Tooth identification (ID) was fitted as a random effect.

### Effects of sodium hypochlorite

Remaining teeth (*n* = 28) were randomly divided into four treatment groups (*n* = 7 per group) subjected to immersion in bleach of varying concentration for differing time intervals as shown in Table 1.

**Table 1:** Treatment groups for study of the effects of bleach on the histological appearance of cementum

**Treatment group**	**Treatment**
1	None - control group
2	Soaked in 4% bleach for 5 min then rinsed
3	Soaked in 4% bleach for 1 min then rinsed
4	Soaked in 2% bleach for 5 min then rinsed

Teeth were cleaned of soft tissue remnants and blood by gentle curettage with a dental scaler then wiped with DNA-free saline. Bleach treatment was applied as per Table 1, followed by rinsing with sterile saline. Sixteen of the teeth (four from each treatment group) were placed into numbered cassettes and prepared for histological examination as described above. Mounted sections were examined at 100×, 200× and 400× magnification using light microscopy, and photographed and qualitatively assessed for the presence or absence of: soft tissue remnants, nuclei in soft tissue remnants, cellular cementum and nuclei in cementum.

Cementum was sampled from the remaining 12 teeth (three from each treatment group) and DNA was extracted as described above.

Quantification was performed using qPCR with SYBR^®^ green chemistry using a previously published 67 bp nuclear target [22]. The qPCR mix consisted of: 5 μL 2× Brilliant II SYBR^®^ green master mix (Agilent Technologies, USA), 0.15 μM forward primer (GGGCAGTGTTCCAACCTGAG), 0.15 μM reverse primer (GAAAACTGAGACACAGGGTGGTTA), 400 ng/μL Rabbit Serum Albumin, 3.3 μL water and 1 μL DNA extract to a total of 10 μL. All samples were run in triplicate including negative (PCR blank) and positive (dilutions of male genomic DNA, Applied Biosystems, USA) controls and extraction blanks. Cycling was performed using a Corbett 6000 Rotogene real-time PCR thermocycler and consisted of an initial 5 min denaturation at 95°C, followed by 45 cycles of 95°C for 10 s, 59°C for 20 s and 72°C for 15 s. Nuclear DNA concentration was determined using the comparative CT method; unknown samples were compared to a standard curve with a range from 0.033 ng/μL to 8.848 ng/μL. This method offers a standard curve with a lower, smaller range for increased sensitivity.

## Results

### Histology

Nucleated cells were observed in abundance in the pulp tissues and in and on areas of cellular cementum. They were also noted in accessory canals, in soft tissue inclusions, and in bone and soft tissue remnants that were present in teeth with constricted furcation areas. No stainable nuclear material was visible within dentine. Cellular cementum was more prevalent at the apical ends of the roots and in the furcation areas. A layer of cementoblasts was observed overlaying some root surfaces. The thickness of cellular cementum varied between teeth and was not uniform on all sides of the same roots. In three out of four teeth the cellular cementum was seen to begin approximately two-thirds up the root but on the fourth tooth it started quite close to the enamel junction. Inter-radicular surfaces displayed, in general, thicker cellular cementum than the outer surfaces of the roots. One tooth displayed a number of highly cellular inclusions in the cementum at the apical end of one root, possibly representing the contents of accessory canals. In another tooth a large number of cells were noted trapped between two closely situated roots. Enamel was not present as it is 96% mineral and was totally removed during the demineralisation process.

### DNA yield from cementum

**Figure 2:**
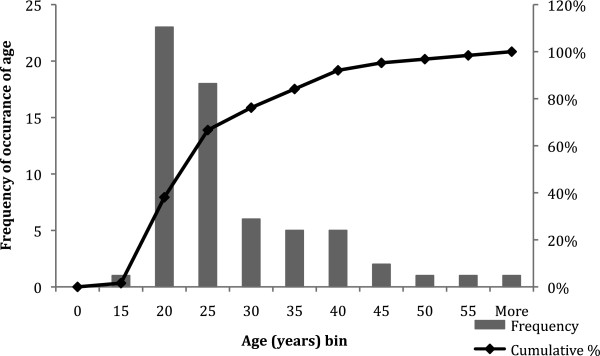
Distribution of chronological age of tooth donors.

Nuclear DNA yield from cementum varied widely between teeth (0.28-173.57 ng/mg, Table 2). The age distribution of tooth donors was biased towards people under the age of 26 years (Figure 2). Seventy-one per cent of donors were aged between 16 and 26 years with every year represented, 17% of donors were aged 29 to 39 years with ages 32 and 38 not being represented, and only 12% of donors were aged between 39 and 60 years with many ages not being represented. The ratio of female to male donors was 59:41. No statistically significant effect was noted of chronological age on DNA yield, although there was a trend showing a decrease in yield with increasing age. Donor sex did not have a statistically significant effect on DNA yield from cementum.

**Table 2:** **Variation in nDNA yield from cementum adjusted for weight of tooth powder sampled (*n*** **= 66)**

**Descriptive statistics (ng/mg)**	
Mean	21.26
SD	26.73
SEM	3.29
Min	0.28
Median	14.42
Max	173.57
95% CI	14.65-27.77

All cementum samples produced sufficient DNA to yield a full STR profile and there were no discrepancies observed between profiles generated from cementum samples and the corresponding reference sample. We found no evidence of contamination and no dropout of alleles was noted.

### Effects of sodium hypochlorite

The control (untreated) group of teeth showed similar histological features to the initial subset of teeth examined. Bleach treated teeth showed a reduction in the presence of and a loss of tissue differentiation in persisting soft tissue remnants in crevices, a reduction in the presence of cementoblasts on the root surfaces, and a reduction in the presence of intact nuclei in the cementum. No structural changes to the cementum were observed.

**Figure 3:**
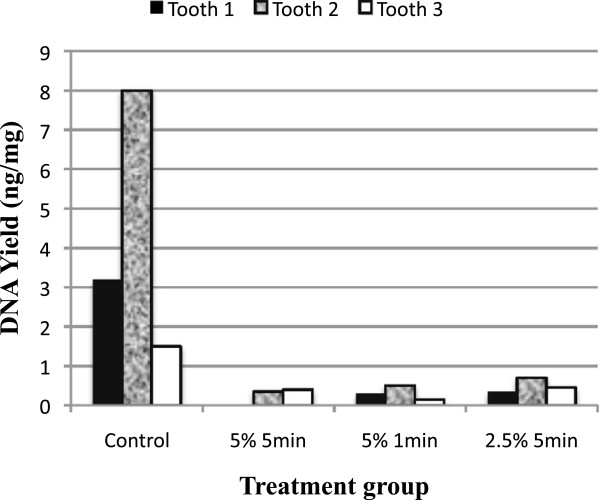
Effects of bleach at varying concentration and exposure time on nuclear DNA yield from cementum.

As seen in Figure 3 the nuclear DNA yields from cementum samples from bleach treated teeth reduced by an order of magnitude at both concentrations (2.5% and 5%) and exposure times (1 and 5 min) in comparison to untreated samples.

## Discussion

In fresh teeth cementum is a reliable source of nuclear DNA, and may be an important and easily accessible source when pulp is absent or compromised. The yield of nuclear DNA from cementum, even after sodium hypochlorite decontamination, suggests that sampling solely from the outer surface of the roots leads to successful STR profiling. Histological examination confirmed that cementum is the only dental hard tissue containing nucleated cells. An absence of visible nuclei in dentine suggests that nuclear DNA recovery from this tissue [8,9] may derive directly from pulp tissue with which it is intimately associated (both developmentally and functionally) or potentially from postmortem cellular breakdown allowing DNA to permeate the dentine mineral.

DNA extraction from teeth for human identification frequently involves non-specific sampling (drilling or whole tooth grinding), requiring specialised equipment, laboratory set-ups and lengthy extraction protocols with large volumes of reagents. These features add significant additional cost, time and complexity to tooth-based DNA identification - a major drawback for disaster victim identification (DVI) and other high throughput identification situations.

Extrapolating our results on fresh teeth to more typical forensic cases involving aged and degraded skeletal remains may not be justified without further research. However, in cases of short postmortem intervals, where human remains are well preserved or in diseased teeth or those from elderly individuals (where pulp is absent or reduced), targeted sampling of cementum as an alternative for DNA analysis and identification offers a number of key advantages. Cementum is readily accessible and easily sampled using manual sampling tools, eliminating the need for specialist equipment to cut, drill and/or grind the teeth thus reducing cross-contamination risks and expense. The DNA extraction process is also simplified and is successful from small sample sizes (15–50 mg) using small volume extraction protocols with the potential for much higher throughput. Cementum contains less mineral than enamel, dentine or even bone, decreasing dependence on EDTA demineralisation steps. In contrast to dentine, DNA recovery from cementum is not adversely affected by dental disease nor age of the individual.

Variation in the abundance and distribution of cellular cementum is to be expected as it is laid down continuously throughout life. In general cementum thickness increases with age [23] but deposition is also affected by functional requirements, the presence of periodontal disease [24] and systemic diseases such as diabetes [25].

Nuclear DNA yields from the cementum of healthy third molar teeth varied by three orders or magnitude (0.28 to 173.57 ng/mg). Previous studies have also shown a large variation in DNA yield from teeth [26,27] but cementum has not previously been examined independently. The reasons for this wide range in DNA yield are unclear. It does not appear to be related to chronological age or sex of the donor, but may be due to variation in the amount of cellular cementum collected from each tooth. Cellular and acellular cementum frequently occur as alternating bands on the tooth and are difficult to distinguish. Despite this, all samples yielded sufficient DNA to produce full STR profiles, confirming the value of targeted sampling of cementum.

External decontamination is often seen as a necessary prerequisite to DNA analysis of postmortem teeth and bones. If teeth are extracted from the jaw under ideal conditions, the value of external decontamination via physical removal or treatment with harsh chemicals needs to be balanced against the negative impact on endogenous DNA recovery. The resistance of teeth to contamination even when post recovery handling is not performed in an ideal manner has been demonstrated [28,29] suggesting that severe decontamination measures may not always be warranted.

Bleach, which dissolves soft tissues and destroys DNA, has been used widely as a DNA decontamination measure in ancient DNA research and forensic DNA practice. Despite this, very little is known about the qualitative or quantitative effects on endogenous DNA in human skeletal remains, including teeth. As an important source of nuclear DNA in the hard tissues of teeth, it is important to understand the effects of bleach on cementum. Histological examination of teeth treated with bleach revealed a reduction in the amount of cellular material visible on the outer surface of the root and a loss of tissue definition in remaining soft tissue remnants. This is consistent with previous observations showing that bleach dissolves soft tissue, with effects related directly to concentration, volume and exposure time [30]. Loss of visible nuclei on the root surface, and in the outer layers of the cementum was also observed suggesting an overall loss of nuclear DNA from cementum and associated sources.

Quantification of DNA yield from bleach treated teeth showed an order of magnitude decrease in comparison to non-bleach-treated teeth. Other studies examining the effects of bleach have studied bone and did not quantify the effects on the endogenous DNA yield [14,17]. These studies also primarily focused on ancient samples, which potentially differ from samples of a forensically significant time span. In younger samples it would be expected that not all the available endogenous DNA would be bound up in protective mineral aggregates. Salamon et al. [14] included several 'modern’ bones in their study and noted DNA outside of the crystal aggregates that was potentially affected by bleach treatment but did not quantify the DNA loss or explore this in any detail. The structural and chemical difference between bones and teeth prevent extrapolation of observations of the behavior of one of these tissues to the other. In our study DNA from cementum treated with bleach was sufficient, in nearly all instances, to produce full STR profiles despite the 10-fold reduction in DNA yield. However, it should be noted that these teeth were healthy fresh samples. In degraded samples it would be expected that the starting amount of DNA would be considerably lower but also that the DNA might be bound to the tooth mineral. Further investigation using degraded teeth of varying postmortem intervals would help understand the true impact of various bleaching regimes on cementum. Potentially teeth at different stages of postmortem decay will display not only differences in their DNA/mineral relationship but also in their porosity influencing the depth of penetration and subsequent effects of bleach. Dissing et. al. [31] demonstrated in fossilised teeth that bleach penetrated through to the pulp chamber. No studies on the porosity of teeth or depth of penetration of bleach have been conducted on more modern samples.

Grinding the tooth surface has also been reported as an alternative or additional method of decontamination. This method can potentially remove all the available cellular cementum which has been reported to have a maximum thickness in upper molars of 25–1140 μm and 20–700 μm in lower molars [32]. Cellular cementum is generally thickest on molar teeth and is predominantly found at the root tips and between the roots [32]. The histological data from this study supported this distribution pattern and demonstrated an increase in cellularity in areas where the cementum was thickest.

## Conclusions

We confirmed that pulp and cellular cementum provide the primary sources of nucleated cells in teeth and demonstrated that cementum is an excellent and easily accessible source of nuclear DNA. Targeted sampling of cementum may be useful in DVI situations where large numbers of individuals need to be sampled quickly, in recently deceased individuals or well preserved remains where specialist laboratory set-up and equipment for sampling and grinding whole teeth are not available, or from diseased teeth or those from elderly individuals where pulp is absent or reduced. Cementum is easily removed from teeth using a scalpel, no special equipment is required and the majority of the tooth is left intact. Cementum samples alone provided sufficient DNA to obtain full STR profiles from all of the teeth examined without a prior decalcification step in the extraction process. Decontamination with bleach reduces the yield of DNA recovered from cementum, which may have a significant effect on STR profiling success of degraded teeth. Tooth extraction from the jaw under controlled conditions may reduce the need for root surface removal or treatment with bleach. However, situations may arise when this is not possible or the teeth available for sampling are no longer in the jawbone. In these cases, the need for more stringent decontamination may be required but should be carried out mindful of possible impacts on DNA in the cementum.

## Abbreviations

DVI: Disaster victim identification; EDTA: Ethylenediaminetetraacetic acid; ID: Identification; PCR: Polymerase chain reaction; qPCR: Real time polymerase chain reaction; RFU: Relative fluorescence unit; STR: Short tandem repeat; UV: Ultra violet.

## Competing interests

The authors declare they have no competing interests.

## Authors’ contributions

DH with assistance from JA, JK and GT conceived and designed the study. DH performed all laboratory work, compiled the data, and performed initial interpretation of results. TH performed formal statistics. DH, with assistance from JA, wrote the manuscript. All authors critically revised the manuscript for intellectual content and approved the final manuscript.
